# Highly Stretchable High‐Performance Silicon Nanowire Field Effect Transistors Integrated on Elastomer Substrates

**DOI:** 10.1002/advs.202105623

**Published:** 2022-01-29

**Authors:** Xiaopan Song, Ting Zhang, Lei Wu, Ruijin Hu, Wentao Qian, Zongguang Liu, Junzhuan Wang, Yi Shi, Jun Xu, Kunji Chen, Linwei Yu

**Affiliations:** ^1^ National Laboratory of Solid‐State Microstructures School of Electronics Science and Engineering Collaborative Innovation Center of Advanced Microstructures Nanjing University Nanjing 210093 P. R. China

**Keywords:** elastomers, field effect transistor, silicon nanowire integration, stretchable electronics

## Abstract

Quasi‐1D silicon nanowires (SiNWs) field effect transistors (FETs) integrated upon large‐area elastomers are advantageous candidates for developing various high‐performance stretchable electronics and displays. In this work, it is demonstrated that an orderly array of slim SiNW channels, with a diameter of <80 nm, can be precisely grown into desired locations via an in‐plane solid‐liquid‐solid (IPSLS) mechanism, and reliably batch‐transferred onto large area polydimethylsiloxane (PDMS) elastomers. Within an optimized discrete FETs‐on‐islands architecture, the SiNW‐FETs can sustain large stretching strains up to 50% and repetitive testing for more than 1000 cycles (under 20% strain), while achieving a high hole carrier mobility, *I*_on_/*I*_off_ current ratio and subthreshold swing (SS) of ≈70 cm^2^ V^−1^ s^−1^, >10^5^ and 134 ‐ 277 mV decade^−1^, respectively, working stably in an ambient environment over 270 days without any passivation protection. These results indicate a promising new routine to batch‐manufacture and integrate high‐performance, scalable and stretchable SiNW‐FET electronics that can work stably in harsh and large‐strain environments, which is a key capability for future practical flexible display and wearable electronic applications.

## Graphical abstract

High‐performance Si nanowire FETs are integrated upon elastomer, within a discrete device‐on‐island architecture, that can sustain large stretching to 50% or repetitive >1000 cycles (@20% strain), with hole carrier mobility, *I*_on_/*I*_off_ current ratio, and subthreshold swing of 70 cm^2^ Vs^−1^, >10^5^ and 199 mV decade^−1^, respectively, paving the way for batch‐manufacturing highly stretchable displays and wearable electronics.

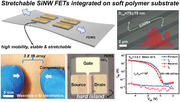

## 1 Introduction

**Figure 1:**
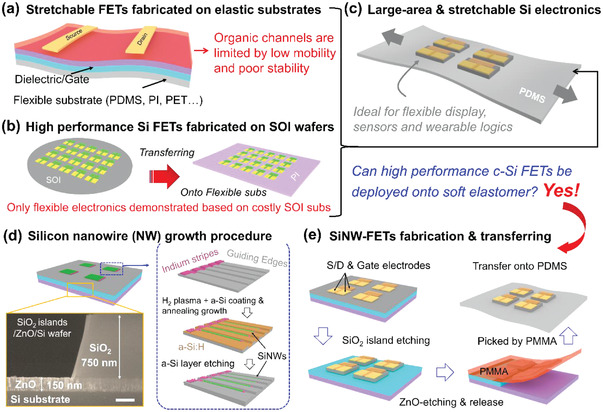
a) Schematic illustrations of the soft FETs with organic channel materials, dielectric layers, and electrodes usually printed on elastomer substrates, compared with b) the fabrication of high‐performance c‐Si FETs on SOI wafers, and then transferring a flexible (not stretchable) substrate. c) depicts schematically the integration of c‐Si FET logics upon discrete hard islands distributed on soft substrates for stretchable display, logic, or sensor applications. d) The typical fabrication procedure of the self‐aligned SiNW array, via IPSLS mechanism, upon 750 nm thick SiO_2_ islands upon a sacrificial ZnO layer (preserved for etching and releasing, see the SEM image in the bottom‐left inset). e) Transferring and device fabrication steps of the SiNW‐FETs upon stretchable PDMS thin film substrate.

Stretchable electronic devices with high electronic performance and durable mechanical elasticity are highly desirable for exploring a wide range of advanced soft or skin‐attached electronics,^[^ ^1^, ^2^, ^3^, ^4^ ^]^ sensors,^[^ ^5^, ^6^, ^7^, ^8^ ^]^ and logics.^[^ ^9^, ^10^ ^]^ Usually, intrinsically stretchable organic thin films, deposited via spin‐coating or printing, have been widely used as the channel material to fabricate field‐effect transistor (FET) devices.^[^ ^11^, ^12^, ^13^, ^14^ ^]^ However, as depicted schematically in **Figure** **1**a, these organic FETs are typically limited by relatively low carrier mobility (compared to their inorganic counterparts), and have poor air‐exposure stability that necessitates extra and less stretchable encapsulation layer protection.^[^ ^15^, ^16^ ^]^ In parallel, inorganic one‐dimensional (1D) nanomaterials have been widely used for developing various high‐performance and flexible electronic devices due to their high mobility, low synthesis cost, and rather high aspect‐ratio 1D geometry that enables good flexibility, low leakage current, and low power consumption.^[^ ^17^, ^18^, ^19^, ^20^, ^21^, ^22^ ^]^ However, in order to achieve stretchability, these 1D NWs need to be assembled as random percolation network,^[^ ^19^, ^22^ ^]^ or buckled into out‐of‐plane 3D wrinkles by attaching to pre‐strained elastomer substrate,^[^ ^23^, ^24^ ^]^ which are difficult to achieve high‐density integration on large area planar substrates.

Alternatively, high‐performance and stable c‐Si microelectronic logic can be first fabricated upon silicon‐on‐insulator (SOI) substrates, then released and transferred, by etching the buried oxide layer, onto flexible polyimide (PI) thin film substrate,^[^ ^25^, ^26^, ^27^ ^]^ as depicted schematically in Figure 1b.^[^ ^28^, ^29^ ^]^ Unfortunately, the high cost and small size of the SOI wafer substrates are still obstacles for establishing large area and low‐cost electronics, such as flexible displays and stretchable sensors and logic.^[^ ^30^, ^31^, ^32^ ^]^ Recently, a similar strategy has been developed to transfer 1D carbon nanotube (CNT) FETs upon soft PDMS substrates, where discrete CNT FETs are placed and protected by discrete PI islands, of 800 ×  800 µm wide and ≈2 µm, with double‐sides encapsulation.^[^ ^1^ ^]^ However, the electronic transport performance has been greatly limited by the randomly crossed CNT network channels, while the overall stretchability is only <20%. Indeed, integrating orderly NWs, particularly high‐quality SiNWs, as the beneficial 1D channels on elastomer substrate for highly stretchable electronics has never been explored so far.

In order to batch‐fabricate ultrathin (*D*_nw_ < 80 nm), high quality and, more importantly, self‐positioned orderly SiNW array, we have developed, in our previous works, a relatively new in‐plane solid‐liquid‐solid (IPSLS) growth mechanism,^[^ ^33^, ^34^, ^35^, ^36^, ^37^, ^38^ ^]^ where indium (In) catalyst droplets can be guided by pre‐defined edge lines to produce planar SiNWs at precise locations, by consuming precoated amorphous Si (a‐Si) precursor layer on substrate surface. Though high‐performance SiNW‐FETs have been successfully demonstrated,^[^ ^37^, ^39^, ^40^, ^41^, ^42^, ^43^ ^]^ based on the high crystallinity 1D channels grown at a rather low temperature <350 °C, a stretchable integration of these SiNW FETs onto soft elastomer thin film substrate, within a hard‐island‐protection architecture, has not been investigated. In this work, we report an orderly growth integration of slim SiNW channels, which can be reliably batch‐transferred onto large area PDMS elastomers upon discrete SiO_2_ islands, as depicted schematically in Figure 1c. The SiNW‐FETs can sustain large stretching strains up to 50% and repetitive testing for more than 1000 cycles (under 20% strain), while achieving a high hole carrier mobility, *I*_on_/*I*_off_ current ratio, and subthreshold swing (SS) of ≈70 cm^2^ V^−1^ s^−1^, >10^5^ and 134 ‐ 277 mV decade^−1^, respectively, and work stably in an ambient environment over 270 days without any passivation protection, providing a solid basis to explore/integrate more advanced stretchable display, wearable electronic and sensor applications.

## 2 Results and Discussion

As schematically depicted in Figure 1d, the SiNWs were first grown via IPSLS mechanism, upon 750 nm thick SiO_2_ islands upon a sacrificial ZnO layer of 150 thick. Specifically, the guiding edges with a depth of approximately 120 nm were first prepared on SiO_2_/wafer substrate via standard photolithography and inductively coupled plasma (ICP) etching. Then, In stripes were defined and evaporated at the ends of the guiding edge lines, prior to being loaded into the plasma enhanced chemical vapor deposition (PECVD) system for a H_2_ plasma treatment, which reduces the thin In_2_O_3_ surface layer and allows them to aggregate into discrete In droplets. After that, a thin film of a‐Si was coated as a precursor at 150 °C, followed by a vacuum annealing at 350 °C that activated the In droplets to absorb the nearby a‐Si and produce aligned SiNWs along the guiding edges. In the end, the remnant a‐Si layer was selectively etched off by H_2_ plasma at 180 °C.

The preparation and transfer process of SiNW FET devices is diagrammed in Figure 1e. The SiNWs were then oxidized at 850 °C for 15 min to form a thin layer of SiO_2_ of ≈10 nm thick. Then, the source and drain electrodes (Pt/Au) were prepared via electron beam evaporation (EBE) and lift‐off procedure, followed by the deposition of SiN*_x_* gate dielectric layer by using PECVD and the preparation of top gate (Pu/Au) electrodes. The hard SiO_2_ islands of 130 µm wide were patterned by using ICP techniques, covered by polymethyl methacrylate (PMMA) layer, and released by HCL etching of the bottom ZnO layer. Then, the released PMMA layer, holding the FETs/oxide islands, was picked and transferred to the PDMS substrate, before being dissolved/removed by acetone solution. More experimental details and explanations were provided in the Experimental Section.

**Figure 2:**
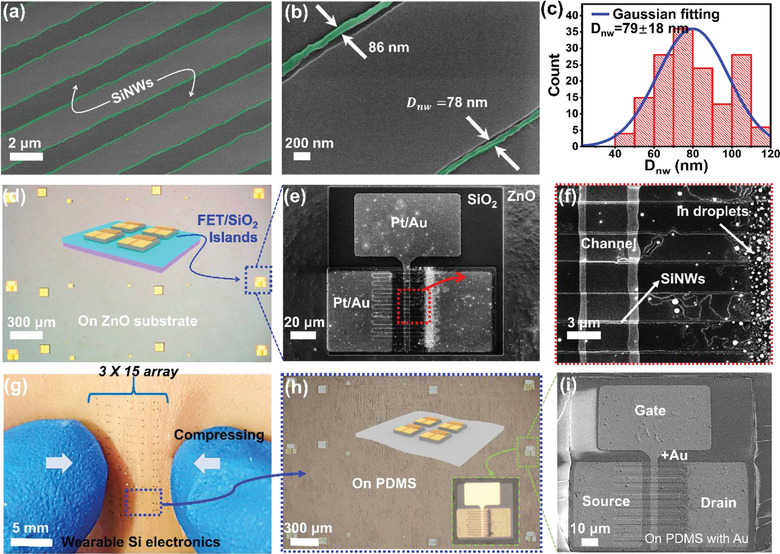
a) A typical scanning SEM image of the edge‐guided SiNWs, with a close examination and diameter statistics provided in b) and c), respectively. d) Microscope image of the SiO_2_ hard islands (with FET units fabricated on top) upon ZnO (sacrificial layer)/wafer substrate, with a schematic illustration of the device configuration provided in the inset. e) and f) provide enlarged SEM characterizations of the FET units on top of SiO_2_ islands, where only the slim guided SiNWs away from the In stripe edges are used as the channels. g) Optical images of the array of the SiNW‐FET/island devices transferred to PDMS thin film and attached to human skin, while close scrutiny of the discrete islands, by using microscope and SEM, are presented in (h) and (i), respectively.

**Figure 3:**
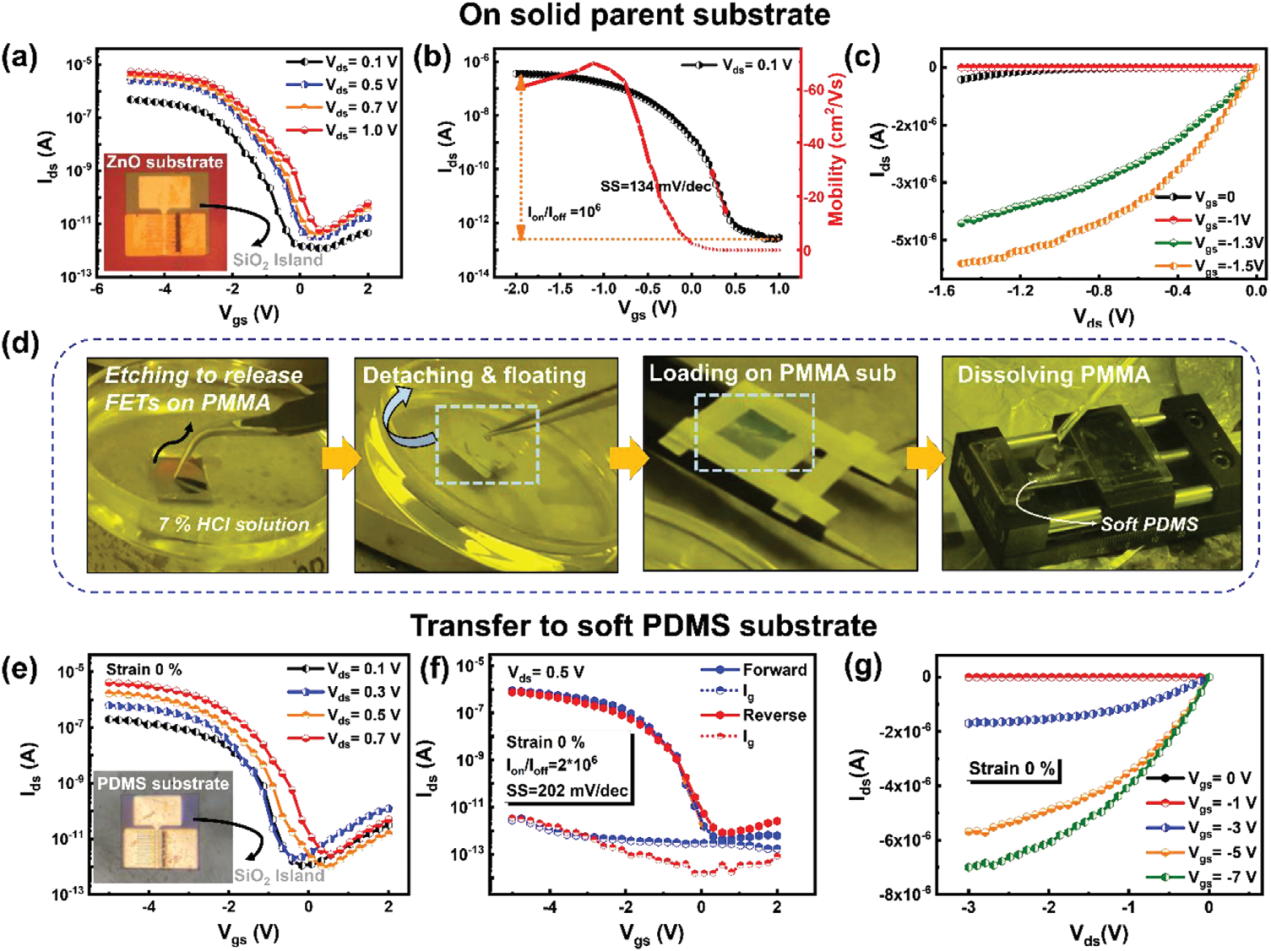
a–c) Electrical transport performances of the SiNW‐FETs on a parent wafer substrate. (a) shows the transfer curves under different biases. The optical image of a hard island FET device on the ZnO (sacrificial layer)/wafer substrate provided in the inset. (b) A typical transfer characteristic (black) of a SiNW FFT, at *V*_ds_ = 0.1 V, where the hole mobility is plotted against *V*_gs_ (red). (c) The output characteristics. d) The specific fabricated process of the SiNW‐FETs was batch‐transferred to soft PDMS thin film. e–g) Electrical transport performance of the SiNW FETs on soft PDMS substrate. (e) shows the transfer characteristic curves under different *V*_ds_ biases. The inset shows the optical image of a hard island FET device on a soft PDMS substrate. (f) The transfer characteristics and hysteresis curves measured under *V*_ds_ = 0.5 V. (g) The output characteristics.

**Figure** **2**a shows the typical field emission scanning electron microscope (SEM, Zeiss Sigma) image of the as‐grown SiNWs along the pre‐defined step edge lines, where slim and orderly SiNWs, tinted to green for the ease of observation, are always found along the guiding edges, with precise orientation/number control and a quite uniform diameter of *D*_nw_ = 79 ± 18 nm, as witnessed in the close SEM view in Figure 2b and the statistics in Figure 2c. Figure 2d provides an optical microscope image (top view) of the as‐fabricated SiNW‐FFT unit on SiO_2_ islands (130 µm wide) located upon sacrificial ZnO (150 nm) layer on a wafer substrate, with a structural configuration as diagrammed in the center inset. Magnified SEM examinations of a selected unit, highlighted by the dashed boxes, are provided in Figure 2e,f, revealing the layout of the source, drain, and gate electrodes of Pt/Au (5/55 nm) and the channel region of *L*_ch_≈3 µm long, composed of a group of ≈16 parallel SiNWs. Note that only the SiNWs segments ≈15 µm away from the In stripe edges are chosen to serve as the channel region, so as to avoid the unguided, random, and thicker SiNWs usually found close to the In stripe edges. Then, the as‐fabricated SiNW‐FET array can be batch‐transferred to soft PDMS thin film, via a procedure described later in **Figure** **3**d, and attached to human skin, as seen in Figure 2g, where an orderly matrix of 3 × 15 FET units on PDMS can be conformally placed upon soft skin and sustain large squeezing distortion without delamination or breakage. An optical microscope image of the transferred SiNW‐FFT devices on PDMS is shown in Figure 2h, where the position and integrity of the discrete islands are found to be very well preserved. This can be further confirmed by closer SEM examination of the transferred FET unit in Figure 2i, after coating with a 2 nm Au layer to enhance surface conductivity, which indicates that the large electrode pads are still complete and crack‐free upon the soft PDMS surface, with the protection of SiO_2_ hard island.

Figure 3a–c shows the typical transfer and output characteristics of the SiNW‐FETs fabricated on the parent solid substrates, showing a p‐type transfer property due to the incorporation of In atoms into c‐SiNW during the planar growth.^[^ ^39^, ^44^ ^]^ A transfer curve measured under *V*_ds_ = 0.1 V bias is extracted and replotted in Figure 3b in logarithm *y*‐axis, with a high *I*_on_/*I*_off_ ratio of ≈10^6^ and a steep SS of 134 mV dec^−1^. The field‐effect hole mobility of the SiNW‐FETs was also calculated to be 70 cm^2^ V^−1^ s^−1^, by using the formula d*I_ds_/*d*V_gs_* = *μ*(*C*/*L* ^2^)*V*, where *μ* stands for the hole mobility, *L* is the length of SiNW channel (*L* = 3 µm), *C* = *Sε/t_ox_* is the gate‐channel capacitance, with *ε*, *S* and *t_ox_* for the dielectric constant, the total channel area and the thickness of SiN*_x_* dielectric layer, respectively. In addition, there is almost no hysteresis observed in the forward and backward scans, as witnessed in Supporting Information Figure S1. In the next step, the SiNW‐FETs were transferred to PDMS thin film, via a procedure as diagrammed in Figure 1e. As showcased in the corresponding photo images in Figure 3d, the sample was first spin‐coated with a PMMA layer (≈200 nm), which holds the SiNW‐FET/island units while the bottom ZnO layer is etched off in 7% HCl solution; Second, the SiNW‐FET/islands were picked and transferred to the surface of PDMS elastomer, followed by an acetone solution dipping to dissolve the PMMA layer; Finally, the SiNW‐FETs were mounted on a unidirectional stretching platform for mechanical and electric testing. Figure 3e–g shows the electrical transport properties of the SiNW‐FET devices, after being transferred to PDMS substrates (without stretching). Overall, the SiNW‐FETs transferred to PDMS can still preserve a high *I*_on_/*I*_off_ current ratio of ≈2 × 10^6^, a reasonable SS of 202 mV dec^−1^ and basically no hysteresis, highlighting the reliability and integrity of the hard‐island‐protected SiNW‐FET transferring procedure.

**Figure 4:**
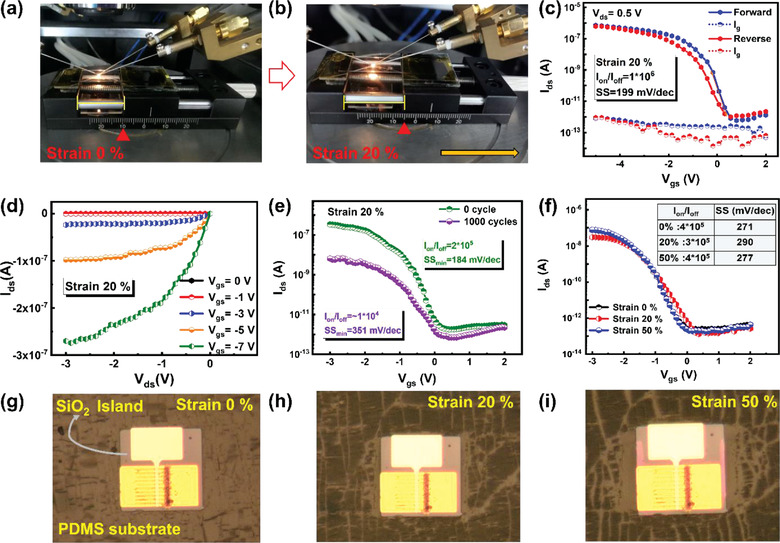
Photographs of the SiNW‐FET device mounted on stretching platform, at a) initial 0% and b) 20% stretching status. c) The transfer characteristics and hysteresis curves measured under *V*_ds_ = 0.5 V at strain 20%. d) The output characteristics curves measured under strain 20% at different gate biases. e) The transfer characteristics curves measured at strain 20% over 1000 cycles. f) The transfer characteristics curves measured at strain (0%, 20%, 50%). g–i) The corresponding enlarged photo images of the hard island region at the different strains on PDMS film.

**Figure** **4**a,b presents the photo images of the testing platforms of the SiNW FET/islands/PDMS under initial 0% or 20% stretching strains, respectively. Actually, the integrity of the SiO_2_ islands under different stretching stains upon PDMS thin film has been systematically testified, as shown in Figure S2a–h (Supporting Information), where it is found that the hard SiO_2_ islands, with different sizes but the same thickness of 750 nm, can sustain large stretching up to 30% without any crack or obvious deformation. Under stretching to 40%, the rectangle hard islands of rectangular began to wrinkle slightly, and further stretching to 60% leads to the formation of tiny cracks in the wrinkled regions. When being stretched to ≈70%–90%, many more cracks emerged first at the corner regions of large pieces, followed by a gradual spreading into the whole islands causing a complete fracture. Note that, small island pieces are found to be more resilient to cracking under large stretching. These observations provide a practical guide for us to choose/design suitable island size/thickness, as a reliable platform to deploy the SiNW‐FET units upon the soft PDMS thin film. For the specific rectangle island design of 130 µm wide used in this work, Figure S2i–k (Supporting Information) shows a series of enlarged images of the initial, 30% stretched and recovered oxide islands, revealing that the island by itself can be rather stable and recoverable after large strain.

Figure 4c–f provides the electrical transport characterizations of the SiNW‐FETs measured under different stretching strains. Overall, the SiNWs FETs/island/PDMS displayed quite stable device performance under stretching strain to 20%, as evidenced by the transfer and output curves shown in Figure 4c–d, where a high *I*_on_/*I*_off_ ratio of ≈10^6^, SS of 199 mV dec^−1^ and low hysteresis of ≈0.2 V can be achieved under 20% tensile strain. More specific transfer characteristics and hysteresis characteristics at different *V*_ds_ (≈0.1–0.7 V) under stretching 0% and 20% are provided in Figure S3, Supporting Information. After 1000 times 20% stretching, as shown in Figure 4e, the *I*_on_/*I*_off_ decreases to ≈ 1×10^4^, SS decreases to 351 mV dec^−1^, this may be caused by the formation of tiny cracks on the soft PDMS substrate after repetitive stretching that degrade the device performance. The *I*_ds_–*V*_ds_ curves measured under strain 0% and strain 20% are provided in Figure S4 (Supporting Information), indicating that the curves before and after stretching almost completely coincide with each other. In addition, the SiNW‐FETs can also be stretched further to 50%, with basically the same transfer characteristic, as seen in Figure 4f and the inset table comparing the extracted On/Off current ratios and SS factors under different stretching strains. This excellent mechanical and electronic performance stability can be assigned to the unique hard‐island protection, as witnessed in the microscope images of the SiNW‐FET/island units on the soft PDMS under gradually increased tensile strain up to 50% in Figure 4g–i. Strikingly, despite the apparent and largely distorted crack marks that appeared on the surrounding PDMS surfaces (due to the formation and fracture of a thin hard SiO_2_ layer on the surface of PDMS upon lasted exposure in air^[^ ^45^ ^]^), the rigid island loaded with FET unit remains basically intact, without any discernable cracking or wrinkle deformations.

**Figure 5:**
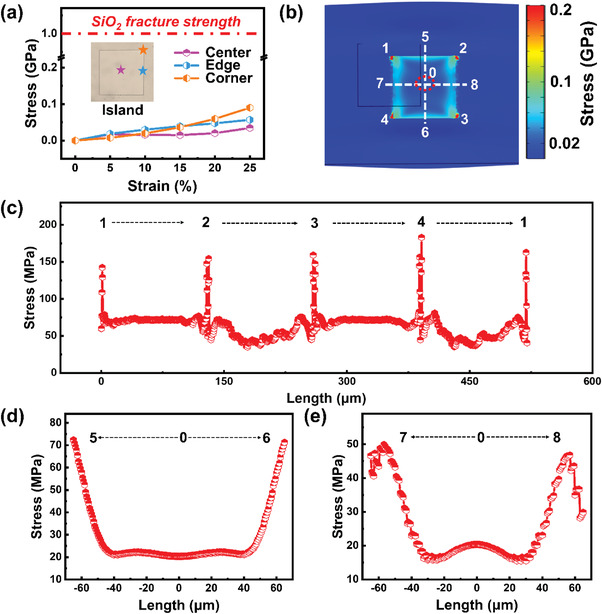
a) Stresses at various locations (center, edge, corner) on a hard island change with strain. The optical image of a SiO_2_ hard island on the PDMS substrate is provided in the inset. b) The strain distribution results of the SiO_2_ hard island under applied strains are between 0 and 20%. The numbers represent the locations of various points on the island. c) The stress changes at four corners of the island during the process of 20% PDMS stretching. d,e) The stress changes from the middle to both sides of the island during strain 20% on PDMS substrate. (d) Vertical stretching direction. (e) Parallel stretching direction.

In order to estimate and understand the strain distribution on the rigid island upon stretched elastomer substrate, finite element analysis (FEA) simulation has been carried out for a simple squared SiO_2_ island, of 130 µm wide and attached to a soft PDMS surface (See the Experimental Section for more details of the model setup and simulation parameters). Under different tensile strains of ≈0%–25%, imposed in the *x*‐axis direction, the local stresses are extracted at three feature points, that is the center, edge, and corner, and plotted against the applied strain in **Figure** **5**a. With the increase of the stretching strain, the stresses at these typical points all increase monotonically. Under the largest strain of 25%, the Corner point experiences the largest stress of 0.1 GPa, but this is still much lower than the fracture strength of ≈1 GPa for typical SiO_2_ thin film.^[^ ^46^, ^47^ ^]^ Interestingly, the stresses found at the edge and center points are even lower, as seen in the stress mapping in Figure 5b and the replotted stress profiles in Figure 5c–e along the labeled pathway, indicating a well mechanical protection of the SiNW channels located at the very center of the island region. Note that, the stresses on the surrounding soft PDMS layer are much lower, compared to those accumulated along the island borders, and thus are almost invisible in the linear stress distribution plot in Figure 5b.

Compared to the flexible or stretchable FETs reported in the literature, as summarized in **Table** **1**, the high‐performance SiNW‐FET/island devices can survive large stretching strains up to 50%, while achieving a high hole carrier mobility of ≈70 cm^2^ V^−1^ s^−1^, *I*_on_/*I*_off_ current ratio of ≈10^6^ and a small SS down to 134 mV/dec, as well as excellent durability that can sustain 1000 stretching (under 20% strain). More importantly, these SiNW channels can be batch‐manufactured via a low‐temperature guided‐growth into pre‐known locations, ready for scalable device fabrication, transferring, and integration upon soft elastomer substrates. All these capabilities combined provide a solid basis to explore a new technological routine to integrate c‐Si electronics for a wider range of soft electronic applications.

**Table 1:** Comparison of SiNW FETs on PDMS substrate to the other flexible/stretchable FET devices in the literature

Channel Materials	Fabrication	Substrate	Tensile Strain [%]	*I*_on_/*I*_off_	SS [mV dec^−1^]	Mobility [cm^2^ V^−1^ s^−1^]	Refs.
IDTBT film	Spin‐coating and transfer	PDMS	0	2.5 × 10^4^	≈6000^a)^	≈0.9	[11]
			100	4.1 × 10^4^	≈6000^a)^	≈0.4	
OSC NW	Electrospinning and transfer	PDMS	0	≈10^3^ ^a)^	≈300^a)^	≈1.0	[12]
			100	≈10^3^ ^a)^	≈300^a)^	≈0.3	
PSHT fibers	Electrospinning and transfer	Elastomer	0	10^5^	≈500^a)^	18	[13]
			70	10^5^	≈500^a)^	18	
Graphene	CVD and transfer	PDMS	0	≈14^a)^	≈2300^a)^	1188	[17]
			5	≈14^a)^	≈3000^a)^	1188	
Graphene	CVD and transfer	PET	Only bending	≈10^a)^	≈1500^a)^	203	[18]
Random SnO_2_ NWs	CVD and transfer	PDMS	0	≈10^6^	500	≈100	[19]
			40	≈10^6^	500	≈100	
In_2_O_3_+5% PVP polymer	Solution and spin‐coating	AryLite polyester	Only bending	≈10^5^	≈350^a)^	10.9	[48]
Carbon nanotube (CNT)	Spin‐coating and transfer	PDMS	0	>10^5^	≈1250^a)^	4.5	[1]
			20	>10^5^	≈1250^a)^	4.5	
CNT	Printing	PUA	0	10^3^ ‐ 10^4^	≈500^a)^	≈30	[49]
			50	10^3^ ‐ 10^4^	≈500^a)^	≈30	
Ordered SiNWs	EBL and transfer	Epoxy polymer	/	>10^5^	300	100	[25]
Random SiNWs	MACE etching and transfer	Polyimide	0	1.2 × 10^7^	/	177	[26]
			1.7	1.2 × 10^7^	/	177	
Si nanoribbon	Solution etching and transfer	Polyimide	Only bending	10^6^	1000	631	[27]
Self‐aligned SiNWs	IPSLS growth and transfer	PDMS	0	1 × 10^6^	134	70	This work
			20	1 × 10^6^	199	70	
			50	4 × 10^5^	277	70	

^a)^ Values estimated from the plots or graphs in the references.

Finally, in view of achieving a scalable device integration upon soft thin film substrates, the discrete SiNW‐FET/island units can be interconnected by conductive and elastic organic/polymeric materials,^[^ ^50^, ^51^ ^]^ or by adopting highly conductive and more stable alloyed silicide NWs springs.^[^ ^52^ ^]^ The latter is particularly suitable for realizing a high‐density integration of discrete logic or LED units upon elastomer substrate. Furthermore, the semiconducting SiNW channels, by themselves, could also be engineered into elastic spring forms and transferred directly onto PDMS substrate for device fabrication,^[^ ^34^ ^]^ similar to the stretchable wavy c‐Si sheets,^[^ ^53^ ^]^ ultra‐long Si nanoribbons^[^ ^54^, ^55^ ^]^ and ultra‐long SiNWs,^[^ ^23^, ^56^ ^]^ which represent a promising avenue to accomplish fully stretchable and durable high‐performance soft electronics, based on the mature c‐Si technology.

## 3 Conclusion

We report here a scalable integration of stretchable SiNW‐FET devices upon elastomer thin film, where the orderly SiNWs were first grown into pre‐designed locations via an IPSLS mechanism, and then transferred and protected upon properly designed rigid SiO_2_ islands on PDMS substrate. Remarkably, the SiNW‐FETs achieve impressive high performance, with a high *I*_on_/*I*_off_ (≈10^6^), low SS (<200 mV dec^−1^), and excellent hole mobility of ≈70 cm^2^ V^−1^ s^−1^, and can survive large stretching strain up to 50% and for 1000 times (at 20% strain). These results indicate a promising routine to integrate high‐performance and reliable c‐Si nanoelectronics onto soft polymer substrate for developing a new generation of wearable/stretchable electronics, sensors, and display with extremely low fabrication cost and excellent mechanical/electronic stability.

## 4 Experimental Section

### Growth of Self‐Aligned SiNWs Array

First, a sacrificial layer of ZnO ≈150 nm thick was deposited on the Si substrate by using magnetron sputtering, and then ≈750 nm thick SiO_2_ was deposited on the ZnO layer in the PECVD system at 300 °C. (The choice of 150 nm ZnO sacrificial layer represents a trade‐off to minimize the deposition and etching durations and to guarantee a quick releasing of the rigid islands during the HCl etching step. The total SiO_2_ layer thickness has to be sufficient/enough to allow for the etching of guiding edges, roughly ≈120 nm into the oxide layer, and good protection of the integrity of the island under large stretching.) Second, after a series of standard photolithography and ICP etching techniques, the guiding edges with a depth of approximately 120 nm were prepared on SiO_2_ surface. Then, the Indium stripe was deposited at the end of the guiding edges by lithography, EBE evaporation, and lift‐off procedure, with the length, width, and thickness of 65 µm, 7 µm, and 13 nm, respectively. Third, the sample was loaded into PECVD system, where a H_2_ plasma treatment at 250 °C for 3–5 min to remove the native oxide layer on the surface of the In particles, with gas flow rate, chamber pressure, RF voltage, and RF power of 15 sccm, 140 Pa, 15 V and 10 W. After that, the 15 nm thick a‐Si thin film was coated at 150 °C for 4 min, with gas flow rate, chamber pressure, RF voltage, and RF power of 5 sccm, 20 Pa, 14 V, and 2 W, respectively. In the following step, the growth was activated in a high vacuum at 350 °C for 1 h. The catalyst In droplets move along the guiding edges and absorbed the a‐Si layer to produce crystalline nanowires behind. Finally, the remnant a‐Si layer can be preserved or selectively removed on SiO_2_ substrate by using H_2_ plasma etching at 150 °C for 6 min with a typical gas flow rate, chamber pressure, RF voltage, and RF power of 15 sccm, 140 Pa, 15 V, and 20 W, respectively.

### Fabrication of Stretchable Si FET on PDMS Substrates

The SiNWs were then oxidized at 850 °C for 15 min to form a thin layer of SiO_2_ of ≈10 nm thick. The source and drain (S/D) electrodes were patterned by lithography, and then the oxide layer of the SiNWs surface was removed by using a 4.0% HF solution for 25 s. After that, the Pt/Au (5/55 nm) (S/D) electrodes were deposited by an e‐beam evaporator and lift‐off procedure. Then, a SiN*_x_* layer (≈20 nm) was deposited as a gate dielectric in the PECVD system at 300 °C, followed by evaporation of Pt/Au (5/55 nm) as gate electrode by using EBE and SF_6_ plasma etching to open via‐holes upon the S/D electrodes in SiO_2_ substrate. Before the FET devices were transferred onto a flexible PDMS substrate, the SiO_2_/hard island was fabricated by using ICP etching. The sample was transferred onto a thin layer of PDMS substrate by PMMA (≈200 nm), and then treated with acetone solution to release the devices. Specifically, a layer of PMMA was formed on the surface of the FET device by spin coating (2000 rpm for 50 s), and dried at 180 °C for 5 min. After that, the 7% HCl solution was used to dissolve the ZnO sacrificial layer and release hard island devices. The FET device fabrication and transfer process were shown in Figure. 1e. The fabricated stretchable Si field‐effect transistor electronics were transferred to a stretchable platform, and electrical performance was characterized with a high precision source‐monitor unit (Keithley 2636B, SMU, USA).

### FEA of the SiO_2_ Hard Island

Finite element simulations were used to analyze the stress distribution at various positions of the SiO_2_/island during the strain 20% on PDMS substrate (Figure 5b). The length, width, and thickness of the PDMS substrate used in the simulation were 720 µm, 380 µm, and 5 µm, respectively. Both the length and width of SiO_2_/island were 130 µm and the thickness was 750 nm. One end of the PDMS substrate was fixed and the other end was stretched to achieve a variety of proportions. The Young's moduli of the PDMS and SiO_2_ were 7.5 MPa, and 70 GPa, respectively. The Poisson's ratios of PDMS, and SiO_2_ were 0.49, and 0.17, respectively. In order to simulate the actual situation, the displacement of the stretching side was set to the specified amount, the displacement of the Z‐axis direction was set to 0 µm, and the *X*‐axis direction (horizontal and vertical to the stretching direction) was not restricted. Ensure that PDMS can stretch and narrow during simulation.

## Conflict of Interest

The authors declare no conflict of interest.

## Supporting information

Supporting Information

## Data Availability

The data that support the findings of this study are available from the corresponding author upon reasonable request.
