# Supplementary material for: Highly Stretchable High‐Performance Silicon Nanowire Field Effect Transistors Integrated on Elastomer Substrates
Source: Adv Sci (Weinh). 2022 Jan 29;9(9):2105623. doi: 10.1002/advs.202105623 (PMC8948590; doi:10.1002/advs.202105623)
Supplement: Supplementary file 1 — Supporting Information [file ADVS-9-2105623-s001.pdf]

ADVANCED SCIENCE Open Access

Supporting Information

for *Adv. Sci.*, DOI: 10.1002/advs.202105623

Highly stretchable high-performance silicon nanowire field effect transistors integrated on elastomer substrates

Xiaopan Song#, Ting Zhang#, Lei Wu, Ruijin Hu, Wentao Qian, Zongguang Liu, Junzhuan Wang, Yi Shi, Jun Xu, Kunji Chen, Linwei Yu**

Supporting Information for

Highly stretchable high-performance silicon nanowire field effect transistors integrated on elastomer substrates

Xiaopan Song[#], Ting Zhang[#], Lei Wu, Ruijin Hu, Wentao Qian, Zongguang Liu[*], Junzhuan Wang, Yi Shi, Jun Xu, Kunji Chen, Linwei Yu[*]

National Laboratory of Solid-State Microstructures/School of Electronics Science and Engineering, Collaborative Innovation Center of Advanced Microstructures, Nanjing University, 210093 Nanjing, P. R. China

[#]These authors contributed equally.

[*]Corresponding E-mails: yulinwei@nju.edu.cn; liuzongguang@nju.edu.cn

.

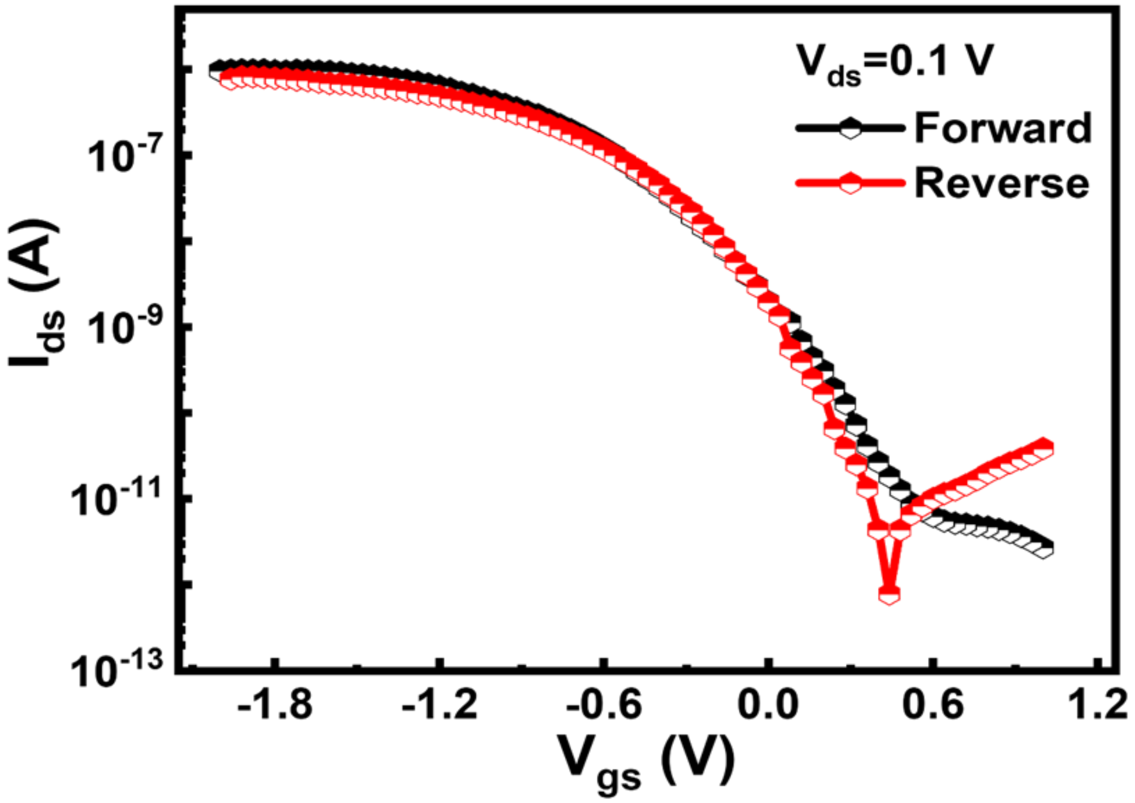

Figure S1. The transfer characteristics and hysteresis curves under V_{ds} =0.1 V on solid parent substrate.

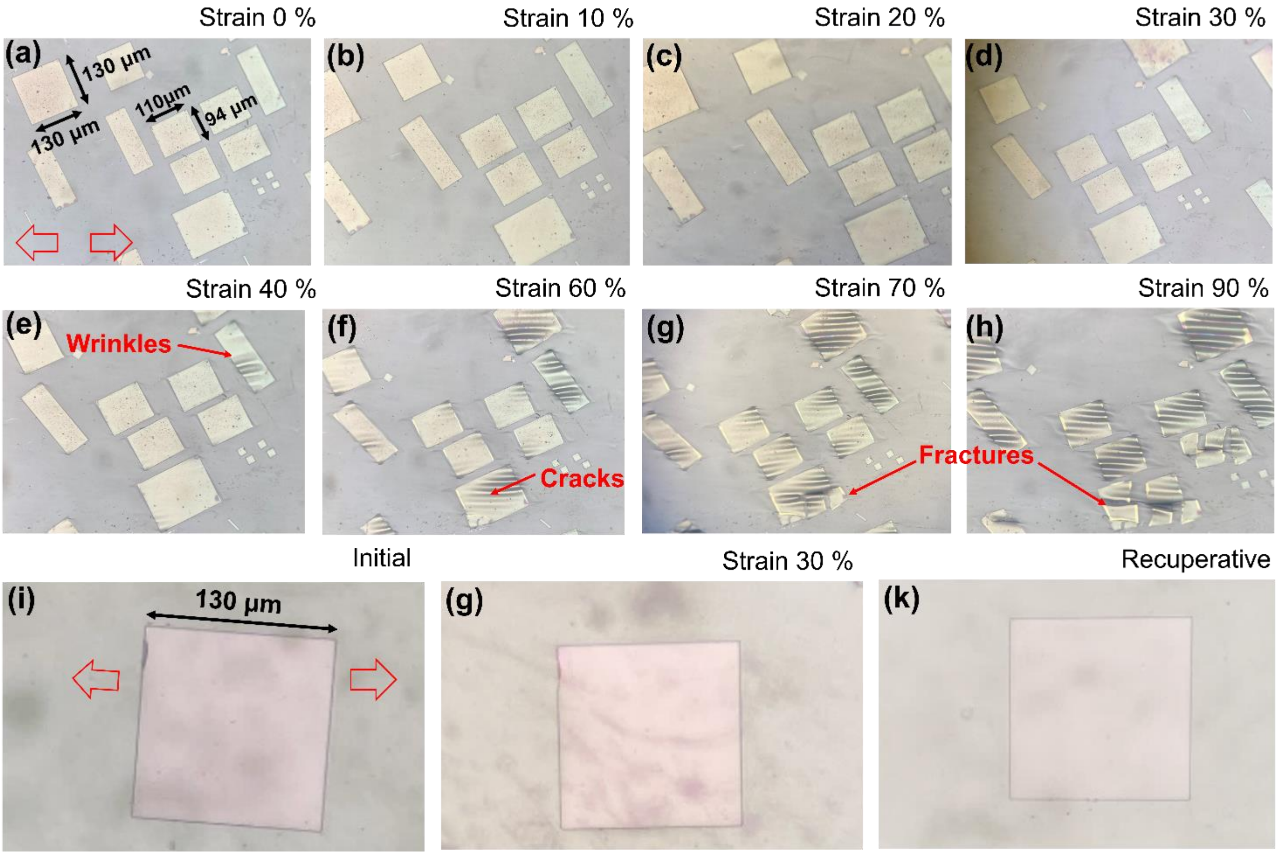

Figure S2. (a-h) The optical microscope image of the SiO_2 hard island of different sizes under applied strains between 0 ~ 90 % on PDMS substrate. (a) Strain 0 %. (b) Strain 10 %. (c) Strain 20 %. (d) Strain 30 %. (e) Strain 40 %. (f) Strain 60 %. (g) Strain 70 %. (h) Strain 90 %. (f-k) Optical image of a SiO_2 hard island with a fixed side length of 130 um under different state. (f) Initial. (g) Strain 30 %. (k) Recuperative.

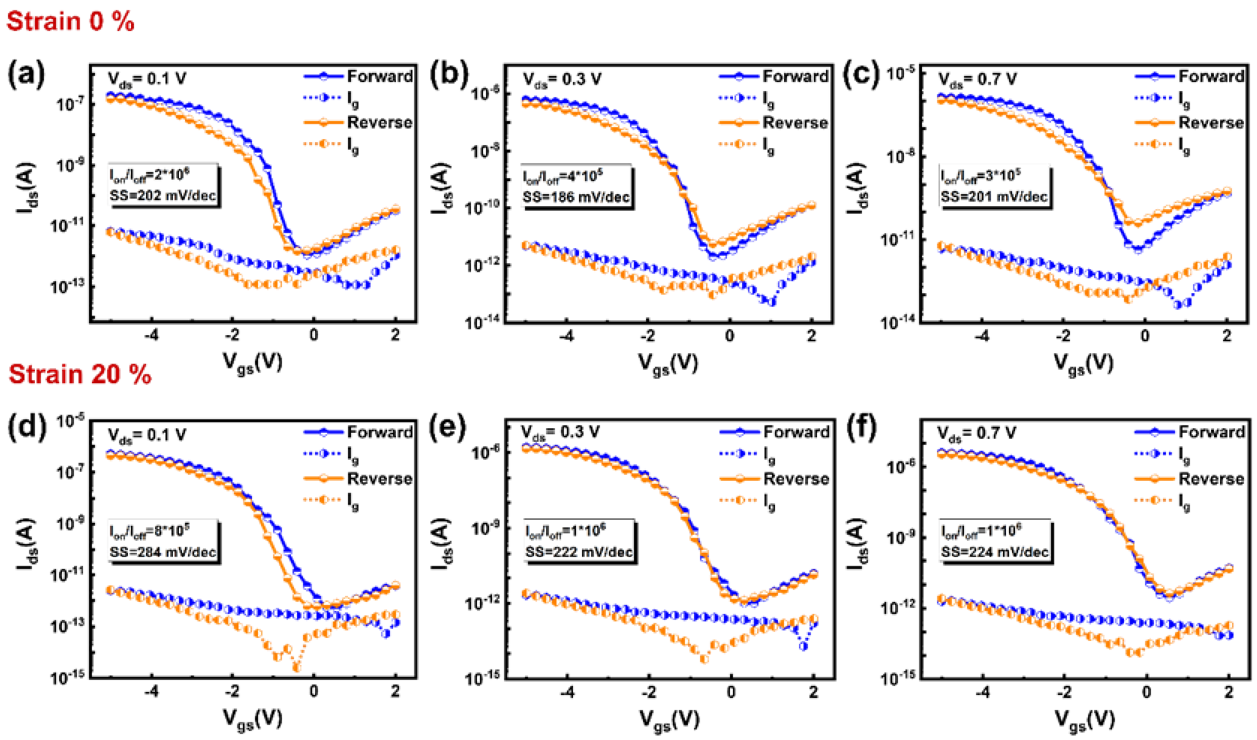

Figure S3. (a-c) The transfer characteristics and hysteresis curves measured under different V_{ds} biases at strain 0 % on PDMS substrate. (a) V_{ds}=0.1 V. (b) V_{ds}=0.3 V. (c) V_{ds}=0.7 V. (d-f) The transfer characteristics and hysteresis curves measured under different V_{ds} biases at strain 20 % on PDMS substrate. (d) V_{ds}=0.1 V. (e) V_{ds}=0.3 V. (f) V_{ds}=0.7 V.

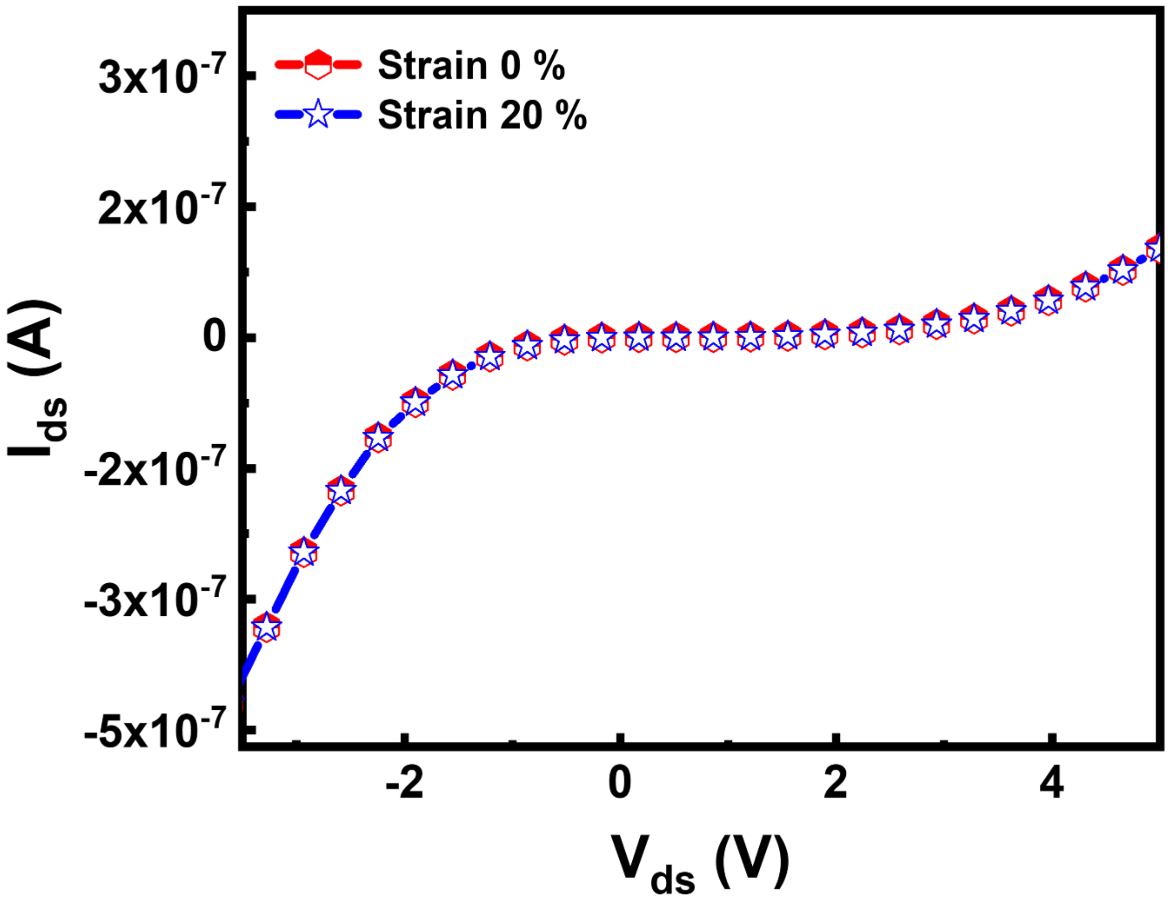

Figure S4. The I_{ds}-V_{ds} curves measured at strain 0 % and strain 20 %.
